# Dynamics of HPV vaccination initiation in Flanders (Belgium) 2007-2009: a Cox regression model

**DOI:** 10.1186/1471-2458-11-470

**Published:** 2011-06-14

**Authors:** Eva Lefevere, Niel Hens, Frank De Smet, Pierre Van Damme

**Affiliations:** 1 Herman Deleeck Centre for Social Policy, Antwerp University, St Jacobstraat 2, 2000 Antwerpen, Belgium; 2 Research Foundation Flanders, Egmontstraat 5, 1000 Brussel, Belgium; 3 Centre for Statistics (CENSTAT), Hasselt University, Agoralaan, Building D, 3590 Diepenbeek, Belgium; 4 Centre for Health Economics Research and Modeling Infectious Diseases (CHERMID), Antwerp University, Universiteitsplein 1, 2610 Wilrijk, Belgium; 5 Medical Management Department, National Alliance of Christian Mutualities, Haachtsesteenweg 579, 1031 Brussel, Belgium; 6 Department of Occupational, Environmental and Insurance Medicine, Catholic University Leuven, Kapucijnenvoer 35/5, 3000 Leuven, Belgium; 7 Vaccine and Infectious Diseases Institute (VAXINFECTIO), Antwerp University, Universiteitsplein 1, 2610 Wilrijk, Belgium; 8 Centre for the Evaluation of Vaccinations, Antwerp University, Universiteitsplein 1, 2610 Wilrijk, Belgium

## Abstract

**Background:**

We investigated dynamic patterns and predictors of HPV vaccination initiation in Flanders (Belgium) by girls aged 12 to 18, between 2007 and 2009, the period immediately after the introduction of the HPV vaccines on the Belgian market. During this period the initiative for vaccination was taken by the girl, her family or the general practitioner/pediatrician/gynecologist.

**Methods:**

We used a Cox regression model with time constant and time varying predictors to model hazard rates of HPV vaccination initiation. The sample existed of 117,151 female members of the National Alliance of Christian Mutualities, the largest sickness fund in Flanders.

**Results:**

The study showed that the hazard of HPV vaccination initiation was higher (1) for older girls, (2) for girls with a more favorable socio-economic background, (3) under more generous reimbursement regimes (with this effect being more pronounced for girls with weak socioeconomic backgrounds), (4) for girls that were informed personally about the reimbursement rules.

**Conclusions:**

When the initiative for HPV vaccination lies with the girls, their families or the physicians (no organized setting) the uptake of the vaccines is affected by both individual and organizational factors.

## Background

On May 2^nd ^2007 the Belgian Superior Health Council (SHC) made its first recommendations regarding HPV-vaccination. It recommended, among others, that organized (i.e. school-based) HPV vaccination should be offered each year to a one-year birth cohort of girls aged 10, 11, 12 or 13 years [1]. In spite of these recommendations, only from September 2010 on school-based HPV vaccination was introduced in Flanders (northern part of Belgium) for girls aged 12 years. Until then, the initiative for vaccination lied with the girls, their families or the physicians (general practitioners/paediatricians/gynaecologists).

In Belgium vaccination of adolescents can take place in- or outside the school-based vaccination system. When vaccines are offered inside the school-based system School Health Services are responsible for monitoring the vaccination status of the adolescents, collecting necessary immunization data, informing parents and children and offering the recommended vaccinations. Parents are free to accept this offer or to get their child vaccinated by a paediatrician, gynaecologist or general practitioner. Vaccines offered to adolescents within the school-based system as well as their administration are free of charge for the vaccinee. When adolescent vaccines are not incorporated in the school-based vaccination system the initiative for vaccination lies with the parents, adolescents or physicians. In that case, the administration costs of the vaccines have to be paid by the vaccinees. For some vaccines the vaccinees have to pay the full price, for others partial reimbursement is provided via the health insurance system. This insurance system is compulsory and covers the entire population. It is organized through private, non-profit sickness funds. The two largest sickness funds are the Christian (National Alliance of Christian Mutualities, henceforth NACM) and the Socialist Mutualities, together insuring about 75% of the population [2]. Two reimbursement regimes exist. If a vaccine is included in the broad benefit package of the "Compulsory insurance" it is (partly) reimbursed by the national government and the reimbursement rates are identical for all sickness funds. A vaccine can also be included in the "Supplementary insurance" of a sickness fund (usually at a lower reimbursement rate than under the Compulsory insurance), a package of supplementary insurance benefits that a sickness fund is free to compose. The reimbursement rates are established by the sickness fund itself, and only hold for its own members.

We studied the dynamic patterns of HPV vaccination initiation in Flanders during the first 2.5 years after the introduction of the vaccines on the Belgian market (January 2007 -June 2009), so before the HPV-vaccines were offered free of charge through the school-based vaccination system. During our period of analysis, the vaccines were partially reimbursed by the health insurance system. The reimbursement regime (Compulsory versus Supplementary insurance) varied over time, mainly depending on the age of the girls (for details see below) and the membership of a respective sickness fund. We used a Cox regression model with time constant and time varying predictors to determine factors affecting the hazard of HPV vaccination initiation.

## Methods

We analyzed data from one sickness fund, the NACM, which covers 53% of the Flemish population. All data extractions and analyses were performed at the Medical Management Department of the NACM under supervision of a medical advisor.

### Sample

We selected girls aged 12 to 18 (years of birth 1989 to 1996) who were member of the NACM on December 31^st ^2006 and who were living in Flanders (N = 151,058). We excluded girls for whom one of the predictor variables (see below) was missing (N = 7,792) and those for whom a vaccine was reimbursed before they had become eligible for reimbursement (N = 1,113), assuming this to be the consequence of inaccuracies in the data. This left us with 142,153 girls.

These 142,153 girls belonged to 117,151 families. Because the similarity in vaccination behavior between sisters could affect the standard errors in our analysis, we randomly selected one girl per family to be included in the study. In this way, the final sample consisted of 117,151 girls.

### Variables

The date of reimbursement of the first dose of a HPV-vaccine was retrieved from the reimbursement claims of the NACM.

Two demographic background variables (year of birth of the girls and province of residence) were available from the membership files of the NACM.

Two predictors reflecting the socio-economic background of the girls were the median income of the neighborhood in which they were living (in quintiles), and a variable indicating whether they were entitled to preferential treatment or not. To construct the first variable we used data from the Belgian Directorate-General Statistics and Economic Information on median net taxable incomes per neighborhood for 2006 (based on tax declarations). We classified each Flemish neighborhood into 1 of 5 income quintiles, linked the address of each girl to the neighborhood, and each neighborhood to the corresponding income quintile. The second variable was the right to preferential treatment. In the Belgian health insurance, certain categories of people enjoy this "preferential treatment", which means they pay lower co-payments. At the time of the analysis, there were two basic grounds for preferential treatment eligibility. First, people receiving certain social benefits (social assistance, guaranteed income for elderly, guaranteed income for disabled), and their partners and descendants, were automatically entitled to preferential treatment. Second, certain categories of people (most importantly: orphans, widows, widowers, disabled, elderly, long term unemployed, children with severe mental or physical illnesses) and their partners and descendants were entitled to preferential treatment after an income test had shown that their household income was below a certain threshold. In both cases, a low household income is a direct or indirect condition for preferential treatment. In our analysis a girl was said to have a right to preferential treatment if at least someone in the family was entitled to this preferential treatment (indicating a low household income) on December 31^st ^2006.

**Figure 1:**
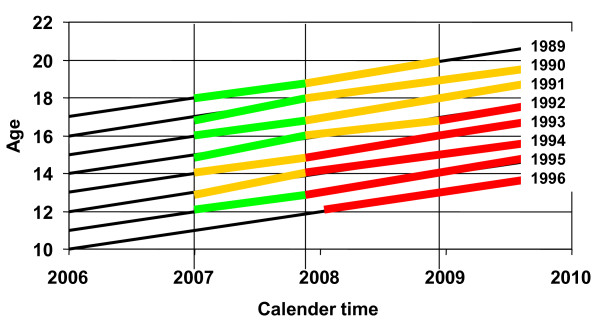
**Lexis diagram illustrating different reimbursement regimes**.

Another variable used in the analysis was the reimbursement regime under which the vaccine was reimbursed. The Lexis diagram (figure 1) illustrates the changes in reimbursement regime over time. The HPV vaccines came on the Belgian market on November 1^st ^2006 (Gardasil^®^) and October 1^st ^2007 (Cervarix ^®^). Between November 1^st ^2006 and January 1^st ^2007 no reimbursement was available. Since we use reimbursement data and not sales data we do not have data on vaccination uptake for this period. Previous research however indicates that the number of vaccines sold in this period was very small [3]. Reimbursement under the Compulsory insurance (red line in figure 1) was offered to girls aged 12 to 15 from November 1^st ^2007 until December 31^st ^2008, and for girls aged 12 to 18 from January 1^st ^2009 on. Reimbursement under the NACM Supplementary insurance was offered to girls aged 13 to 14 from January 1^st ^2007 to October 30^th ^2007 and to girls aged 16 to 19 between November 1^st ^2007 and December 31^st ^2008 ("Supplementary insurance, strict", yellow line in figure 1). Until October 30^th ^2007 the age criteria for Supplementary insurance were sometimes applied flexibly, so that exceptionally, when a member not meeting the criteria asked for reimbursement, this was also granted (in a limited number of cases) ("Supplementary insurance, flexible", green line in figure 1).

A final variable added to the analysis was whether or not the girl had received an information letter on the inclusion of HPV vaccination in the package of Compulsory insurance. When this measure was introduced on November 1^st ^2007, the NACM proactively informed the girls approaching the maximum age for reimbursement (girls born between November 16^th ^1991 and December 31^st ^1992) on the reimbursement rules. In the letter (sent on October 31^st ^2007) objective information about the vaccine and the reimbursement was given. Emphasis was put on the fact that they did not have a lot of time left to get their first vaccine, since no reimbursement would be granted after their 16^th ^birthday.

### Statistical methods

First, we looked at the bivariate associations between HPV vaccination initiation and some background characteristics. The significance of these associations was assessed by means of the (two-sided) chi-square test. For this analysis, HPV vaccination initiation was an indicator denoting whether or not at least one dose of the HPV vaccine was reimbursed to a girl during our period of analysis.

Next, we estimated a Cox regression model with time-constant and time-dependent covariates (SAS procedure PROC PHREG) to model the time to HPV vaccination initiation. HPV vaccination initiation was then the age (in days) at which the first HPV vaccine dose for a girl was reimbursed. It was calculated as the number of days between the date of reimbursement of the first dose of a HPV vaccine and the date of birth of the girl. Year of birth of the girl, province of residence, preferential treatment and median income of the neighborhood where the girl was living were added as time-constant variables. Three time-varying variables were added. The first two indicated the reimbursement regime, with the Compulsory insurance as the reference category and the two other regimes (Supplementary insurance strict and flexible) as time varying indicators taking on value 1 in periods when the regime was in place and value 0 otherwise. The second was the receipt of a NACM letter. For each girl born between November 16^th ^1991 and December 31^st ^1992, this variable was an indicator taking value 1 in the period from November 1^st ^2007 to the sixteenth birthday of the girl and value 0 at other times. For the other girls in the analysis, the indicator took on value 0 over the whole period of analysis. Because it was possible that the cost of the vaccines and the NACM letter had a different impact on HPV vaccination initiation by people from different socio-economic backgrounds [4,5] we added two interaction effects: one between the type of reimbursement regime (i.e. Supplementary insurance (flexible and strict taken together) versus NIHDI reimbursement) and the right to preferential treatment, and one between receipt of the NACM letter and the right to preferential treatment.

Following the suggestion by Therneau and Grambsch periods in which a girl was not at risk (either because the vaccine did not exist or because the girl was not eligible for reimbursement) were treated as missing [6].

The results of our Cox regression are expressed in Hazard Ratios (HR). A HR indicates the relative likelihood of HPV vaccination initiation in two groups of people with different values of a predictor at any given point in time. It can also be interpreted as the likelihood that a girl in the group with higher hazard initiates HPV vaccination first [7], an interpretation that is intuitively easier to understand.

## Results

### Descriptives

**Figure 2:**
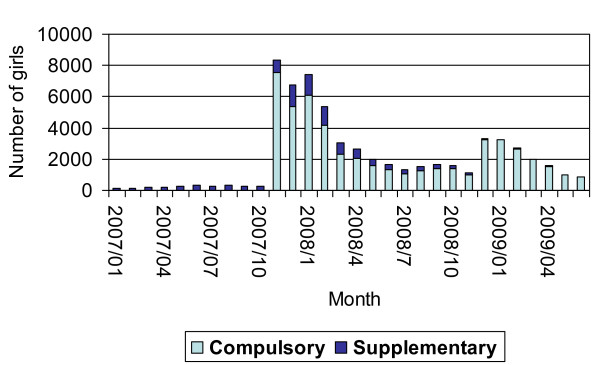
**Number of girls starting HPV vaccination per month and per reimbursement regime**.

From January 1st 2007 until June 30th 2009 61,550 girls (53%) out of our sample started with HPV vaccination. Figure 2 shows the number of girls starting HPV vaccination per month and per reimbursement regime. Three periods can be discerned. During the first period (only reimbursement via the Supplementary insurance) the number of girls starting with HPV vaccination remained relatively low. Then, when reimbursement via the Compulsory insurance was introduced, this number spiked, after which it very slowly decreased again. In this period most of the vaccines were reimbursed under the Compulsory insurance, but the number reimbursed under the NACM Supplementary insurance also increased. Finally, when the reimbursement under the Compulsory insurance was extended to older girls, the number of girls starting HPV vaccination again spiked. Meanwhile the Supplementary insurance gradually became unnecessary.

Table 1 presents the number and percentage of girls that had initiated HPV vaccination between January 2007 and June 2009 according to some (time constant) background characteristics. We found a clear social gradient, with girls having a right to preferential treatment or living in a neighborhood with low median income having a much smaller probability for having started HPV vaccination. Further, vaccination uptake slightly differed according to the province of residence. Finally, there were large and significant differences in vaccination initiation between girls of different years of birth, with girls born in 1992 having the highest vaccination uptake (82%) and girls born earlier or later having gradually decreasing uptake percentages. To interpret these differences, we had to take into account the time varying reimbursement rules and the letter sent by the NACM by means of a Cox regression model.

**Table 1:** HPV vaccination initiation according to background characteristics

		Vaccination initiation N (%)	No vaccination initiation N (%)	P (chi square)
				<0.0001

Year of birth	1989	3,081 (20.2)	12,202 (79.8)	

	1990	8,400 (53.4)	7,342 (46.6)	

	1991	8,270 (53.7)	7,118 (46.3)	

	1992	12,227 (81.9)	2,702 (18.1)	

	1993	10,758 (74.9)	3,615 (25.2)	

	1994	8,959 (65.2)	4,783 (34.8)	

	1995	6,690 (47.9)	7,278 (52.1)	

	1996	3,166 (23.1)	10,560 (76.9)	

Preferential treatment				<0.0001

	Yes	1,816 (33.0)	3,692 (67.0)	

	No	59,735 (53.5)	51,908 (46.5)	

Province				<0.0001

	Antwerp	16,369 (53.0)	14,537 (47.0)	

	Limburg	8,669 (57.2)	6,488 (42.8)	

	East-Flanders	13,466 (50.5)	13,210 (49.5)	

	West-Flanders	12,995 (49.0)	13,520 (51.0)	

	Flemish Brabant	10,052 (56.2)	7,845 (43.8)	

Median income neighborhood^A^				<0.0001

	Quintile1	7,103 (43.0)	9,404 (57.0)	

	Quintile2	11,301 (50.6)	11,019 (49.4)	

	Quintile3	12,411 (53.3)	10,868 (46.7)	

	Quintile4	14,240 (55.1)	11,598 (44.9)	

	Quintile5	16,496 (56.5)	12,711 (43.5)	

^A ^The quintiles are calculated for the whole of Flanders: each quintile encompasses 20% of all tax declarations in Flanders.

### Cox regression model

Table 2 presents the results of the Cox regression. Because the interaction between receipt of the NACM letter and the right to preferential treatment was not significant, it was not retained in the final model. We advance four main findings:

**Table 2:** Cox regression model: Hazard of HPV vaccination initiation

	Hazard ratio	95% CI
Year of birth		

1989	19.39	17.47-21.52

1990	11.74	10.89-12.65

1991	4.32	4.11-4.55

1992 (base)	-	-

1993	0.61	0.55-0.67

1994	0.37	0.33-0.41

1995	0.21	0.19-0.24

1996	0.23	0.20-0.25

Province		

Antwerp (base)	-	-

Limburg	1.13	1.10-1.16

West-Flanders	0.85	0.83-0.87

East-Flanders	0.88	0.86-0.90

Flemish-Brabant	1.00	0.98-1.03

Preferential treatment		

No (base)	-	-

Yes	0.55	0.52-0.58

Median income neighborhood		

Quintile 1	0.75	0.72-0.77

Quintile 2	0.93	0.90-0.95

Quintile 3 (base)	-	-

Quintile 4	1.04	1.02 -1.07

Quintile 5	1.10	1.07-1.12

Reimbursement regime		

Compulsory (base)	-	-

Supplementary strict	0.09	0.09-0.09

Supplementary flexible	0.01	0.01-0.01

Letter NACM		

No (base)	-	-

Yes	2.28	2.08-2.50

Preferential treatment (Yes) * NACM reimbursement (strict or flexible)	0.55	0.47-0.64

First, girls eligible for reimbursement under the Compulsory insurance had a substantially higher hazard of HPV vaccination initiation than girls eligible for reimbursement under the Supplementary insurance. Controlling for other factors, the hazard of starting HPV vaccination under the strict Supplementary insurance was only 9% of the hazard under the Complementary insurance ([HR] = 0.09, 95% CI 0.09-0.09). The hazard of starting HPV vaccination under the flexible Supplementary insurance was even lower: only 1% of the hazard under the Complementary insurance ([HR] = 0.01, 95% CI 0.01-0.01). For people with preferential treatment this effect was stronger, as indicated by the negative sign of the interaction between having a right to preferential treatment and being eligible for Supplementary insurance reimbursement.

Second, the hazard of HPV vaccination initiation was higher for girls with a more favorable socio-economic background. The hazard of HPV vaccination initiation for girls with preferential treatment was only 55% of the hazard for girls without preferential treatment ([HR] = 0.55, 95% CI 0.52-0.58). Likewise, the hazard differed according to the median income of the neighborhood where the girl was living.

**Figure 3:**
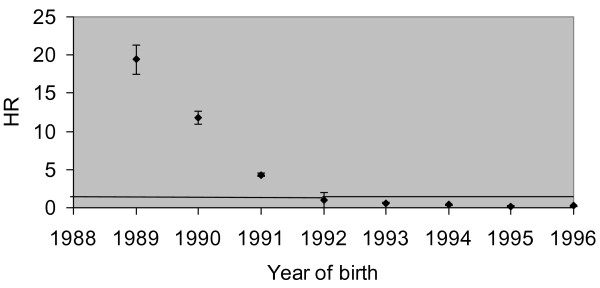
**Cox regression model: hazard ratios with 95% confidence intervals for year of birth illustrating the age-effect**.

Third, the hazard of HPV vaccination initiation was higher for older girls. This is illustrated in figure 3 which shows the hazard ratios for year of birth. The different pattern for year of birth when considering vaccination initiation percentages by June 30^th ^2009 (table 1) or hazard ratios (table 2 and figure 3) illustrates the fact that hazard ratios do not directly relate to vaccination uptake. The percentage girls that initiated HPV vaccination by June 30^th ^2009 (table 1) refers to the situation at one specific moment in time. This situation is the result of all the girls having followed a certain trajectory over time with continuously changing rates of HPV vaccination initiation (hazard rates) depending on the values of the predictors at each moment in time. The high uptake percentage of girls born in 1992 is mainly due to the fact that girls in this age group received the NACM letter. The lower uptake percentage of girls born in 1991 (and before) compared to girls born in 1992 is mainly due to the fact that they never had a right to reimbursement under the Compulsory insurance. The lower uptake percentage of girls born in 1993 (and after) compared to girls born in 1992 is mainly due to the effect of age.

Fourth, girls who were personally informed about the Compulsory insurance reimbursement and the fact that they were approaching the age limit had a substantially higher hazard of HPV vaccination initiation ([HR] = 2.28, 95% CI 2.08-2.50).

### Sensitivity analysis

By introducing a variable in the model that indicated whether a girl had no, one or two or more sisters in the same age group we tested whether having a sister affected the vaccination behavior. Introducing this variable did not significantly change the parameter estimates of the other variables.

## Discussion

The organization of HPV vaccination and local circumstances vary widely between countries, and so does HPV vaccination uptake. Previous large-scale studies using individual level data investigated HPV vaccination uptake in an organized setting, be it through mass vaccination sessions [8] or via school-based vaccination [9-11]. We looked at HPV vaccination initiation in a non-organized setting, whereby the initiative for vaccination lied with the girls, their family or the gynecologist/pediatrician/general practitioner. We focused on vaccination initiation and not on vaccination completion (a complete regimen of the vaccine consists of 3 doses). Note that of the 45,614 girls that started HPV vaccination before November 2008 (leaving them at least 9 months to complete the vaccine regimen within our period of analysis, the recommended interval between the first and the last dose being 6 months) 86% completed the regimen by June 2009.

There can be various reasons why the probability of HPV vaccination was higher under the Compulsory insurance as compared to the Supplementary insurance. Most likely the generosity of the system played an important role. The full price of the HPV vaccines was 130.22 euro per dose (3 doses required to be fully vaccinated). Under the Compulsory insurance, the reimbursement was 119.42 euro per dose (123.02 euro for people eligible for preferential treatment), leaving a co-payment of 10.80 (7.20) euro per dose. Under the Supplementary insurance, the reimbursement was only 50 euro per dose (75 euro in case of preferential treatment). Reducing out of pocket costs has been shown to improve vaccination uptake for various children, adolescent and adult vaccines [4,12-15]. In addition, the administrative procedure of each of the reimbursement regimes might also have been important. Under the Compulsory insurance the girl buying the vaccine only had to pay the non-refundable part of the price of the vaccine. Under the Supplementary insurance the girl had to first advance the full price of the vaccine, after which part of it was refunded. Further, there was also substantially more media attention and advertising (towards girls, their families and medical doctors) for the Compulsory insurance. These might have had an important influence on the uptake of the vaccine: it has been shown that mass media interventions can significantly affect various health behaviors [16].

Vaccination uptake was substantially lower among girls from lower socio-economic backgrounds. Such association between socio-economic status and adolescent vaccination has been found in other studies, too [8,17-22]. Socio-economic differences in HPV vaccination uptake are particularly relevant since people with low SES have a higher risk of contracting cervical cancer [23,24]. An obvious explanation might be the financial barrier caused by the co-payments [4,12-15]. However, most likely other reasons have also played a role, such as a differential influence of media attention on people with different socio-economic backgrounds or advertising being more directed to certain categories of people. Free, school-based HPV vaccination, as organized in Flanders from September 2010 on, might help to overcome this problem. School vaccination programs are indeed considered as the best way to achieve good adolescent vaccination uptake [15]. Evaluating the performance of school vaccination programs in Flanders with regard to socio-economic differences in vaccination uptake however remains difficult: socio-economic differences are found for some of the vaccines currently included in the school vaccination program, too, but these might be partially attributed to differences in the availability of documentation on vaccination at home, since studies have to rely on documentation available at home [25].

Older girls had a substantially higher probability of vaccination initiation than younger girls. While these findings are in line with recent findings from the US [26], in the Netherlands a different relationship between age and HPV vaccination has been found, with both the oldest and the youngest age categories having a slightly lower HPV vaccination uptake [8]. A possible explanation for the age effect we found is an increased parental acceptance of the vaccine as the age of the daughter increases [27]. Another possible explanation is procrastination: if girls had a long period of eligibility for reimbursement before them, they might have been less inclined to start with vaccination.

Finally, the letter by which girls were informed about their eligibility for HPV vaccine reimbursement and the approaching age limit had a large influence on vaccination initiation. This is in line with previous research in which patient reminder and recall systems have been shown to significantly affect vaccination behavior [28].

## Conclusion

We studied determinants of HPV vaccination initiation in Flanders (Belgium). Despite the fact that during our period of analysis the initiative for vaccination lied with the girls, their family or the gynecologists/pediatricians/general practitioners (no organized setting), vaccination uptake was high for certain subcategories of girls. However, large socioeconomic differences were found. This suggests that school-based vaccination, as it was introduced in Flanders in September 2010 (although with the possibility to refuse the vaccine or to get vaccinated by a physician outside the school-based system), might be preferred. Future research could give more insight in the evolution of HPV vaccination uptake under this school-based system, as well as in the factors underlying the observed socioeconomic differences.

## Competing interests

The authors declare that they have no competing interests. PVD acts as chief and principal investigator for vaccine trials conducted on behalf of the University of Antwerp, for which the University obtains research grants from vaccine manufacturers; speakers' fees for presentations on vaccines are paid directly to an educational fund held by the University of Antwerp. PVD receives no personal remuneration for this work.

## Authors' contributions

EL designed the study, jointly developed the statistical model and drafted and reworked the article. NH participated in the design of the study, jointly developed the statistical model and continuously followed-up the analyses. FDS was responsible for the acquisition of the data and the follow-up and supervision of the analyses at the NACM. PVD continuously followed-up the analyses and reworked the article. All authors read and approved the final manuscript.

## Pre-publication history

The pre-publication history for this paper can be accessed here:

http://www.biomedcentral.com/1471-2458/11/470/prepub
